# Sport Readaptation: Where Do We Draw the Lines Between Professionals?

**DOI:** 10.3389/fspor.2019.00062

**Published:** 2019-11-27

**Authors:** Daniel Rojas-Valverde, Juan Carlos Gutiérrez-Vargas, Braulio Sánchez-Ureña

**Affiliations:** ^1^Centro de Investigación y Diagnóstico en Salud y Deporte, Escuela Ciencias del Movimiento Humano y Calidad de Vida, Universidad Nacional, Heredia, Costa Rica; ^2^Grupo de Avances en Entrenamiento Deportivo y Acondicionamiento Físico, Facultad Ciencias del Deporte, Universidad de Extremadura, Cáceres, Spain; ^3^Centro de Estudios para el Desarrollo y Rehabilitación en Salud, Escuela Ciencias del Movimiento Humano y Calidad de Vida, Universidad Nacional, Heredia, Costa Rica; ^4^Programa de Ciencias del Ejercicio y la Salud, Escuela Ciencias del Movimiento Humano y Calidad de Vida, Universidad Nacional, Heredia, Costa Rica

**Keywords:** physical rehabilitation, return-to-play, recovery, sport injury, sport science

## Introduction

Sport is an activity that is in a state of constant and dynamic evolution. These characteristics are essential to ensure its entertainment value. Currently, sport is undergoing significant changes in the way it is managed, trained for, and even competed in. The current trend in sport regarding physical and physiological demands is typified by an increase in the intensity and frequency of competition. This could lead to a disturbance in physical and mental performance due to different external and internal factors (Halson, 2014). In the last decade, high-intensity competition and training (Di Salvo et al., 2009; Bradley et al., 2010, 2011; Carling et al., 2012; Gabbett, 2015) has defined sports performance in many disciplines. Match congested tournaments and periods of high-frequency training and competition in team sports are increasingly common (Dellal et al., 2013; Rojas-Valverde et al., 2018; Birdsey et al., 2019; Pino-Ortega et al., 2019). In endurance events, there is a rise not only in the number of competitions but in the intensity and volume of these events. This could lead to serious future complications that could be avoided by preventive and recovery injury protocols (Chlíbková et al., 2015; Rojas-Valverde et al., 2019; Rubio-Arias et al., 2019; Gutiérrez-Vargas et al., in press).

This dynamic in endurance and anaerobic sports is causing an increase in the amount of injuries and their relapse due, among other things such as repeated high-intensity (Bengtsson et al., 2013; Carling et al., 2016) and long-endurance activity (Small and Relph, 2018; Warrick et al., 2019), to insufficient rest and recovery between exertions. Therefore, nowadays, high performance is achieved according to the capacity to recover better and faster between psychobiological efforts (Bauman, 2005). Effective recovery from training and competitive loads represents the difference between success and failure in sport (Kellman, 2002). That is why the scientific community has been tasked to study various methods of recovery from both physical and mental fatigue (Kellmann et al., 2019). In this sense, optimal protocols are needed in order to maintain an athlete's abilities and determine recovery pathways to maximize performance after fatigue or injury (Fowles, 2006). Additionally, the implementation of appropriate, sensitive, and reliable methods to assess and mitigate fatigue and injuries is needed because it can provide information not only on physical and mental condition but on the prevention and evolution of physical performance during this complex process (Thorpe et al., 2017).

This change in sports dynamics puts high stress on the athlete (Taylor et al., 2012; DiFiori et al., 2014), and it is required that he maintain sport form and recover it quickly when an adverse situation arises (Kellmann, 2010), for example, an injury. This need to recover as soon as possible is based on the fact that there is evidence that if this process is not carried out properly, in an adequate and timely manner, athletes may experience persistent functional impairment of the injured structure (Orchard and Best, 2002; Maniar et al., 2016) and reduced functional capacity to perform sport-specific movement (Silder et al., 2010a; Fyfe et al., 2013), and could lead to adaptive changes in the locomotor patterns of sport-related movements (Silder et al., 2010b; Sole et al., 2012).

During the treatment of an injury, the athlete receives attention from several providers such as medical doctors, physical therapists, psychologists, nutritionists, coaches, and sport scientists (Kraemer et al., 2009). These professionals are responsible for guiding the athlete through different phases of the return to activity before full medical clearance is given. At some point in the recovery process, once there is no pain, the athlete enters a stage where the objective is the improvement of proprioception, coordination, strength, and endurance and initiates certain specific actions involved in the sport to train without danger of relapse. This transition becomes essential because, despite the fact that the athlete has recovered from a medical point of view, preparation for competition requires the restoration of the physical qualities mentioned above (Kraemer et al., 2009). This sport-specific training may be beyond the qualifications or knowledge of those who cover his medical needs (Walsh et al., 1999). Due to the aforementioned, the need arises within sports clubs for a figure termed a training physician (Walsh et al., 1999) within the body of support for the athlete. This professional is currently called a sport readaptator and will facilitate the transition between the disappearance of pain after an injury and recovery until the athlete returns to regular competitive activity (Jiménez-Rubio et al., 2019).

Considering that almost all of the professionals involved in injury rehabilitation processes are limited in their abilities to drive an athlete from acute injury to recovery (Pabian et al., 2011) and that each professional staff member generally thinks they have the best capacity to make decisions in this injury process (Shrier et al., 2014), it is necessary to clarify each professional's role. Additionally, due to the incorporation of a new professional figure who is necessary for adequate recovery from injuries, the objective of this document is to clarify, based on scientific evidence, the role of professionals involved in the sporting process after an injury, specifically analyzing the new figure of the sports readaptator and, based on this, to propose a workflow for this type of intervention for the best and fastest recovery of the athlete. The above is fundamental to avoid interference between the functions of professionals, always protecting the constant coordination between the health providers of the athlete.

## The Injury Recovery Process: Who and When?

**Figure 1:**
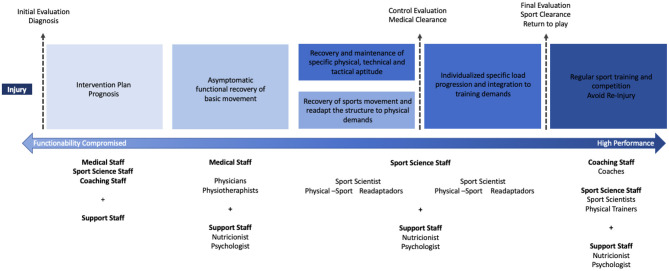
Flow of interdisciplinary and multidisciplinary approach to sport injury recovery.

The coordination between professionals during the recovery process from a sports injury is essential. For this reason, efforts have been made previously to define the functions of each professional involved in this process as well as the different phases in which they come into play (Kraemer et al., 2009; Paredes and Martínez-De Haro, 2009; O'Brien et al., 2019). Knowing that there are gray areas regarding the competences and roles of each professional in different arenas, a review of the literature was carried out in order to deal with the definition of certain areas such as sports medicine, physiotherapy, sports science, training, nutrition, psychology, and readaptation. A description of the role and functions of each professional is needed to clarify the contribution of each one during a sports injury, understanding that the process must be a complement to the implementation and practice of each discipline's knowledge. Figure 1 presents a proposed flow for an interdisciplinary and multidisciplinary approach to sports injury recovery.

### Sports Physicians

Also called sport medical doctors or sports medicine physicians, these professionals are in charge of medical treatment, drug administration, and doping control and prevention (Hoberman, 2002; Dikic et al., 2013). All surgical interventions are in their management area, as are imagological diagnosis and biochemical tests. Their role in sport injury is to give the initial diagnosis and medical treatment and, if surgical intervention is necessary, make a major contribution to the medical clearance decisions (Kraemer et al., 2009).

### Physiotherapists

Also called sport therapists, their role in sport injury recovery is to use certain methods and tools in order to reestablish asymptomatic functional movement in basic patterns. These professionals aim to restore motion, neuromuscular control, balance, and reflex control and to facilitate pain management, limit swelling, and protect injured structures (Kraemer et al., 2009; Arvinen-Barrow et al., 2014).

### Sport Scientists

Also known as athletics trainers, performance staff, conditioning, and strength trainers, their function is to restore strength, endurance, most basic physical performance functions, and specific sport locomotion and actions and to reestablish competitive performance functions (Patel and Baker, 2006; Järvinen et al., 2007; Kraemer et al., 2009). After medical clearance, their work will focus on programming and prescribing individualized sport-specific load and gradually integrating the athlete into regular training demands. Once sport clearance is achieved, this professional should prepare athletes for the demands of high-level competition and help them avoid re-injury. The recovery from fatigue between efforts is also an area of action for these professionals.

### Coaches

The main objective of the coach is to enable their athletes to obtain the highest performance possible (Ojala and Thorpe, 2015). They play fundamental roles during training and competition, such as in motivation, education, organization, planning, and mentoring (Short and Short, 2005). They have to be able to analyze scientific data and translate it into practice in their coaching and training programs. The aim of coaches during injury recovery is to track the development of the condition and to reinsert the player into technical and tactical team training and competition.

### Nutritionists

As a primarily objective, they are in charge of providing proper nutrition during training, competition, and recovery according to the particular energy demands of different activities. Currently, they are essential in the control of ergogenic aids to achieve recovery between loads or injury. The above includes advising athletes about their diet, nutritional supplements, and fluid intake (Maughan and Shirreffs, 2012).

### Psychologists

Sport psychologists analyze mental and motivational factors that could limit sporting performance. In a recovery program, psychological factors are essential, and fear and apprehension are common issues during the injury process (Walker et al., 2007). Psychologists should be active in the recovery process, addressing situations during the rehabilitation program such as insecurity when performing exercises that could remind the athlete of the injury mechanisms, when an opponent approaches at high speed, or when any other hazardous conditions are perceived (Erickson and Sherry, 2017).

### Physical and Sport Readaptators

This new professional must lead the reconditioning phase (Dhillon et al., 2017), understood as the transition that takes place once the rehabilitation of the injury is almost complete and when the athlete is ready to begin strength and conditioning activities. Their main objective is to readapt the injured structure to support physical demands through sport-specific exercise prescription and the recovery of sport-specific locomotion. Additionally, physical readaptadors should support the maintenance of specific physical, technical, and tactical aptitudes during the rehabilitation process.

Despite the fact that there are clearly defined competencies for each professional involved in the recovery from injuries in sport, based on fundamental principles (Piggott et al., 2019), it is essential to acknowledge that interdisciplinary approaches are needed to achieve an effective and efficient recovery. The involvement of diverse disciplines toward a common goal provides a better understanding of the complex phenomenon of injury and could lead to a better overview of the challenges of the recovery process, as well as a better comprehension of the factors that could influence the return-to-play process. Furthermore, it is important to emphasize that the diagnosis, prognosis, intervention plan, sport clearance to final return-to-play decisions, and re-injury prevention must be taken as a consensual determination among all professionals as an intervention group, each of them assuming their role and contributing their knowledge for the sole purpose of preserving the health of the athlete.

With regard to physical rehabilitation, due to the lack of or little need to create a new professional figure, there is legislation in some countries that gives this function to professionals in sport science and human movement science. In these cases, sport science professionals are assigned the function of physical and sport readaptator by law due to their knowledge regarding the recovery of the physical condition of injured athletes (Campos-Izquierdo and Lalín-Novoa, 2012). Additionally, new legislation is complementing professional degrees in sport science with knowledge, procedures, and specific attitudes to physically adapt, recover, or re-educate through physical exercise (Campos-Izquierdo et al., 2010; Campos-Izquierdo and Lalín-Novoa, 2012).

## Conclusion

Athletes' trust in professionals and self-determination and the compromise and communication of all the health providers to the athlete in the process of recovering from an injury is essential to recover the physical and psychological form necessary for return-to-play and receiving the sport and medical all-clear (Kraemer et al., 2009; Podlog et al., 2011). Considering the abilities necessary for the achievement of this objective, such as knowledge of the fundamental components of the prescription of exercise (Kraemer et al., 2002), it has been found that a professional in sports sciences and human movement is the suitable person to execute this essential function within the interdisciplinary team overseeing the recovery of the athlete once the injury is asymptomatic and until they recover their sport-specific strength and conditioning capabilities, leaving those factors related to competition to the respective professionals (Shrier et al., 2014). It is essential to recognize the importance of coordinating with the knowledge bases of other professionals in the area, such as sport medicine doctors, physiotherapists, sport scientist, nutritionists, and psychologists, and to create interdisciplinary injury interventions. This comprehensive approach to the recovery process is the path to success in this type of intervention (Kautz et al., 2007).

## Author Contributions

DR-V conceived and designed the idea for the article and prepared the initial draft. DR-V, JG-V, and BS-U gave substantial revisions, provided critical feedback and helped shape the manuscript and verified the rationale of the paper. BS-U supervised the writing of the manuscript. All authors gave their final approval of the content for publication.

### Conflict of Interest

The authors declare that the research was conducted in the absence of any commercial or financial relationships that could be construed as a potential conflict of interest.
